# The association of dietary insulinemic indices with PI3K, PTEN, and Akt gene expressions in visceral and subcutaneous adipose tissues among individuals undergoing abdominal surgery

**DOI:** 10.3389/fnut.2024.1467686

**Published:** 2024-10-04

**Authors:** Hamid Ahmadirad, Farshad Teymoori, Hossein Farhadnejad, Ghazaleh Shimi, Golaleh Asghari, Emad Yuzbashian, Maryam Zarkesh, Parvin Mirmiran, Alireza Khalaj

**Affiliations:** ^1^Department of Clinical Nutrition and Dietetics, Faculty of Nutrition Sciences and Food Technology, National Nutrition and Food Technology Research Institute, Shahid Beheshti University of Medical Sciences, Tehran, Iran; ^2^Nutritional Sciences Research Center, Iran University of Medical Sciences, Tehran, Iran; ^3^Department of Nutrition, School of Public Health, Iran University of Medical Sciences, Tehran, Iran; ^4^Nutrition and Endocrine Research Center, Research Institute for Endocrine Sciences, Shahid Beheshti University of Medical Sciences, Tehran, Iran; ^5^Department of Cellular and Molecular Nutrition, Faculty of Nutrition Science and Food Technology, National Nutrition and Food Technology Research Institute, Shahid Beheshti University of Medical Sciences, Tehran, Iran; ^6^Department of Agricultural, Food and Nutritional Science, University of Alberta, Edmonton, AB, Canada; ^7^Cellular and Molecular Endocrine Research Center, Research Institute for Endocrine Sciences, Shahid Beheshti University of Medical Sciences, Tehran, Iran; ^8^Department of Surgery, Tehran Obesity Treatment Center, Shahed University, Tehran, Iran

**Keywords:** insulinemic indices, glycemic indices, PI3K, PTEN, Akt, gene expression

## Abstract

**Background/objective:**

The current study investigates the association between dietary insulinemic indices and Akt, PTEN, and PI3K gene expressions in visceral adipose tissue (VAT) and subcutaneous adipose tissue (SAT) among individuals undergoing abdominal surgery.

**Materials and methods:**

This cross-sectional study was conducted on 176 individuals, aged 18–84 years, who had undergone abdominal surgery. The participants were classified based on body mass index (BMI) as normal (BMI < 25 kg/m^2^), overweight (BMI = 25–29.9 kg/m^2^), and obese (BMI ≥ 30 kg/m^2^). The food frequency questionnaire was used to determine dietary glycemic and insulinemic indices. Real-time polymerase chain reaction was conducted for the expression of PI3K, PTEN, and Akt genes.

**Results:**

In the final adjusted model, in normal-weight patients, there was an inverse relationship between the lifestyle with a higher insulinemic potential and the PI3K gene expression in VAT. In addition, there was an inverse association between dietary insulin load and the Akt gene expression in VAT. However, a higher glycemic index was positively associated with the PTEN gene expression in VAT. In overweight patients, a high insulinemic potential of the diet was associated with higher PTEN gene expression in VAT. In obese individuals, there were positive associations between lifestyle index for insulin resistance and hyperinsulinemia and the PI3K gene expression in VAT. Moreover, the higher insulinemic potential of diet and lifestyle was positively related to a higher expression of the PTEN and Akt genes in VAT.

**Conclusion:**

Our findings revealed that high insulinemic lifestyles and dietary patterns may be related to the expression of PI3K, PTEN, and Akt in adipose tissues.

## Introduction

Insulin is one of the most important hormones affecting the different organs in the human body (1) and also plays a main role in energy and macronutrient metabolism (2). Insulin resistance (IR) and hyperinsulinemia are two insulin-related diseases that lead to many chronic diseases (3). Diet and physical activity are considered affective factors in insulin homeostasis and incidents of IR and hyperinsulinemia (4).

In the previous decade, many studies have been conducted to predict the amount of insulin secretion using dietary intakes. At first, glycemic index (GI) and glycemic load (GL) were developed to estimate secretion of the blood glucose after eating food items containing carbohydrates compared to reference food such as bread (5, 6). After that, Prof Jennie Brand-Miller at the University of Sydney developed the insulin index (II) and insulin load (IL) to estimate the increase in serum insulin after consuming each food item (7). In 2016, Tabung et al. developed the empirical dietary index for hyperinsulinemia (EDIH), the empirical lifestyle index for hyperinsulinemia (ELIH), the empirical dietary index for insulin resistance (EDIR), and the empirical lifestyle index for insulin resistance (ELIR) to estimate the potential of diet and lifestyle factors including physical activity and body mass index (BMI) in the IR and hyperinsulinemia among the American population (8). Mokhtari et al. validated and developed the insulinemic indices of diet and lifestyle including the lifestyle index for hyperinsulinemia (LIH), lifestyle index for insulin resistance (LIR), dietary index for hyperinsulinemia (DIH), and dietary index for insulin resistance (DIR) among Iranian adults (9). Previous studies have assessed the association between dietary insulinemic indices and chronic diseases including obesity (10), type 2 diabetes (T2D) (11), cardiometabolic disorders (12), cardiovascular diseases (13), and cancer (14). A prospective cohort study observed a positive association between EDIH and ELIH indices with the occurrence of obesity in Iranian adults (10). Another cohort study found that higher adherence to EDIR, ELIR, and ELIH scores were associated with increased diabetes risk (11). Moreover, a positive association was observed between DIR and LIR scores with cardiometabolic disorders including hypertension, metabolic syndrome (MetS), IR, and T2D (12). Teymoori et al. demonstrated that a greater adherence to the lifestyle and diet with a higher ELIR and EDIR was related to the increased risk of cardiovascular diseases and chronic heart diseases (13). Furthermore, a meta-analysis of prospective cohort studies observed a direct relationship between EDIH and the risk of cancer incidence as well as cancer mortality (14).

Once insulin binds to its receptors on the surface of adipose tissue cells, it activates the PI3K/Akt signaling pathway functions, resulting in increased glucose transporters mainly in the skeletal muscle cells and adipocytes. However, the main function of PTEN is to block the PI3K pathway by dephosphorylating phosphatidylinositol 3,4,5-trisphosphate (PIP3) to phosphatidylinositol 4,5-bisphosphate (PIP2), thus blocking the PI3K signaling cascade (15, 16). Animal and human studies indicated that hyperinsulinemia and IR could change the PI3K, PTEN, and Akt gene expressions and result in metabolic dysfunction (17, 18). Therefore, it seems that examining the relationship between the insulinemic potential of diet and the expression of the mentioned genes could help to identify the role of food patterns in mechanisms involved in the occurrence of metabolic disorders. Several studies assessed the association between feeding patterns and single food groups with the expression of these genes in humans and animals (19–23), e.g., recent studies reported that fruit and whole grains intake activates the expression of the PI3K/Akt pathway (19, 20). However, to the best of our knowledge, the association between dietary glycemic and insulinemic indices and PI3K, PTEN, and Akt gene expressions in visceral adipose tissues (VATs) and subcutaneous adipose tissues (SATs) has not been investigated, and the effect of these indices on the genes involved in the occurrence of these diseases and the pathways that are activated or deactivated by the influence of insulinogenic diet and IR induced are unclear. Therefore, in the current study, we aimed to investigate the possible association of dietary glycemic and insulinemic indices with the expression of genes involved in insulin action such as PI3K, PTEN, and Akt in VAT and SAT (due to differences in insulin sensitivity and glucose homeostasis in these two tissues) among individuals undergoing abdominal surgery.

## Methods

### Study population

In the current cross-sectional study, 176 adults, aged 18–84 years old, who had been admitted for elective abdominal surgery such as umbilical and inguinal hernia repair, were recruited from Mostafa Khomeini and Khatam Al-Anbia Hospitals, Tehran, Iran. The individuals were categorized by their BMI into three groups: 40 normal-weight participants (BMI < 25 kg/m^2^), 31 overweight participants (BMI = 25–29.9 kg/m^2^), and 105 obese participants (BMI ≥ 30 kg/m^2^). We included individuals according to the following criteria: adults with hospitalization less than 3 days, free of any diagnosed diabetes or cancer, not pregnant or lactating, without any lipid-lowering, or anti-obesity medication, and not following any special diets for at least 3 months.

### Ethical statement

All participants filled out a written informed consent. This study was conducted in conformance with good clinical practice standards and was performed according to the Declaration of Helsinki 1975, as subsequent amendments. Furthermore, this study was approved by the Research Ethics Committees of the National Nutrition and Food Technology Research Institute of Iran (Approval ID: IR.SBMU.NNFTRI.REC.1403.036).

### Dietary measurement

Trained interviewers collected the usual dietary intakes of participants through a face-to-face interview using a valid and reliable 168-item semi-quantitative food frequency questionnaire (FFQ) (24, 25). Interviewers asked individuals to state their frequency of intake on a daily, weekly, or monthly basis during the year before the surgery for each food item by selecting one of the following categories: never or less than once a month, 3–4 times per month, once a week, 2–4 times per week, 5–6 times per week, once daily, 2–3 times per day, 4–5 times per day, and 6 or more times a day. Portion sizes of consumed foods, reported in household measures, were then converted into grams. We used the United States Department of Agriculture’s (USDA) Food Composition Table (FCT) to compute the daily energy and nutrient intake (26). Iranian FCT was used for local food items that do not exist in the USDA FCT (27).

### Calculation of dietary insulinemic indices

*Dietary glycemic index and glycemic load* (28): GI = [(carbohydrate content of each food item) × (number of servings/d) × (GI)]/total daily carbohydrate intake; GL = (carbohydrate content of each food item) × (number of servings/d) × (GI); For *dietary insulin index and insulin load* (29, 30): II = IL × 100 ÷ [∑(energy content of food (kcal/serving) × frequency of food consumption (serving/day))], and IL = ∑[Insulin index of food × energy content of food (kcal/serving) × frequency of food consumption (serving/day)] ÷ 100.

*Dietary and lifestyle insulinemic indices* (8, 9).

Details of the development and calculation of insulinemic indices including EDIH, EDIR, EDIH, and EDIR (8) that were introduced by Tabung et al., and also DIH, DIR, LIH, and LIR (9), which were presented by Mokhtari et al., have been described in previous studies. These insulinemic indices are computed based on two groups of food components including positive and negative determinants. The food group components and the weight of each component to the total score insulinemic indices are indicated in Supplementary Table 1.

### Anthropometric and physical activity measurements

Trained dieticians used the standard demographic questionnaire to evaluate age, sex, medical history, and medication use. The body weight and height were measured, and the BMI was computed as weight (kg) divided by the square of height (m^2^). A reliable and validated Persian version of the International Physical Activity Questionnaire (IPAQ) was used to evaluate the physical activity during interviews, and all measurements were expressed as Metabolic Equivalents per week (METs/week) (31).

### Sample collection and quantitative real-time polymerase chain reaction analysis of PI3K, PTEN, and Akt expression

During the surgery, a specialist collected 50–100 mg of VAT and SAT, added them to RNase and DNase-free microtubes, directly froze them in liquid nitrogen, and then moved them to storage at −80°C. TRIzol reagent (Invitrogen, USA) was used to extract total RNA from VAT and SAT according to the manufacturer’s protocol. The quality of extracted RNA was evaluated using a Nanodrop spectrophotometer (ND-1000, USA), and the absorption ratio (260/280 nm) was in an acceptable range. Before the synthesis of complementary DNA (cDNA) using a cDNA synthesis kit (BIOFACT, South Korea) based on the manufacturer’s recommendations, the RNA was treated with DNase I to remove traces of genomic DNA. The product was stored at −20°C for further research. The glyceraldehyde-3-phosphate dehydrogenase (GAPDH) was used as the reference gene for normalization across samples (32, 33). Primers based on the sequences of the National Center for Biotechnology Information (NCBI) GenBank database were checked using the Genrunner Software (version 3.05). Primer sequences of PI3K, PTEN, Akt, and GAPDH were provided in Table 1. Real-time quantitative reverse transcription PCR (qRT-PCR) was conducted using a Rotor-Gene 6000 (Corbett Research, Sydney, Australia) in 20 μL volumes containing 10 μL 2X SYBR Green Master Mix (BioFact, South Korea), 1 μL of the cDNA, 7 μL RNase-free water, 1 μL forward primer, and 1 μL reverse primer.

**Table 1:** Sequences and information of primers.

Genes	NCBI Ref.	Primers’ sequences 5′–3′	Tm	Product size (bp)
PI3K	NM_006218.4	F: CAGAACAATGCCTCCACGA	57	122
		R: CACGGAGGCATTCTAAAGTC		
PTEN	NM_000314.8	F: AAGCTGGAAAGGGACGAACT	59	145
		R: CGCCTCTGACTGGGAATAGT		
Akt	NM_001382431.1	F: CACTTTCGGCAAGGTGATCC	59	94
		R: GTCCTTGGCCACGATGACTT		
GAPDH	NM_001357943.2	F: CTGCTCCTCCTGTTCGACAGT	60	100
		R: CCGTTGACTCCGACCTTCAC		

F, forward; R, reverse.

For all genes, the samples were run in duplicate for inter-assay control along with GAPDH as the reference gene and the non-template control (NTC). Amplification was carried out with the following thermal cycling conditions: 5 min at 95°C for denaturation, followed by 45 cycles at 95°C for 30 s, 60°C for 30 s, and 72°C for 30 s for annealing, amplification, and quantification. The relative expression of PI3K, PTEN, and Akt in each sample was computed using the comparative threshold cycle (Ct) method based on the study conducted by Livak et al. (34) according to the following formula:

$\Delta Ct={Ct}_{\left(\mathrm{PI}3\mathrm{K,PTEN,Akt}\right)}–{Ct}_{\left(\mathrm{GAPDH}\right)}$

$\mathrm{Relative expression}=2^{-\Delta Ct}$

All qRT-PCR laboratory procedures were written based on the MIQE guidelines.

### Statistical analysis

Data analysis was conducted using SPSS, version 21 (Statistical Package for Social Sciences). We used the Kolmogorov–Smirnov test and histogram chart to assess the normality of the data. Qualitative variables were described as frequency (percentages), and quantitative variables were reported as mean ± SD or median (25–75 interquartile range). Relative mRNA expression levels were calculated for each sample separately using internal reference genes GAPDH with the formula 2^−(ΔCt)^, where ΔCt is Ct_(PI3K, PTEN, Akt)_ − Ct _(reference genes)_ (34). Participants were classified into three groups based on their BMI. Differences in variables between the normal weight (BMI < 25 kg/m^2^), overweight (BMI = 25–29.9 kg/m^2^), and obese participants (BMI ≥ 30 kg/m^2^) were compared using the chi-square test and one-way ANOVA test for categorical and continuous variables, respectively. We normalized the data of gene expressions as dependent variables using the RNOmni (Rank Normal Transformation Omnibus Test) package in R software and then used the normalized variables for regression analyses. We used the linear regression to assess the association between the dietary insulinemic indices and the PI3K, PTEN, and Akt expression in VAT and SAT, and unstandardized *β* with a 95% confidence interval (CI) was reported after adjusting for age, sex, and energy intake. A *p*-value <0.05 was used as the statistical evaluation tool.

## Results

### Demographic characteristics of participants

**Figure 1:**
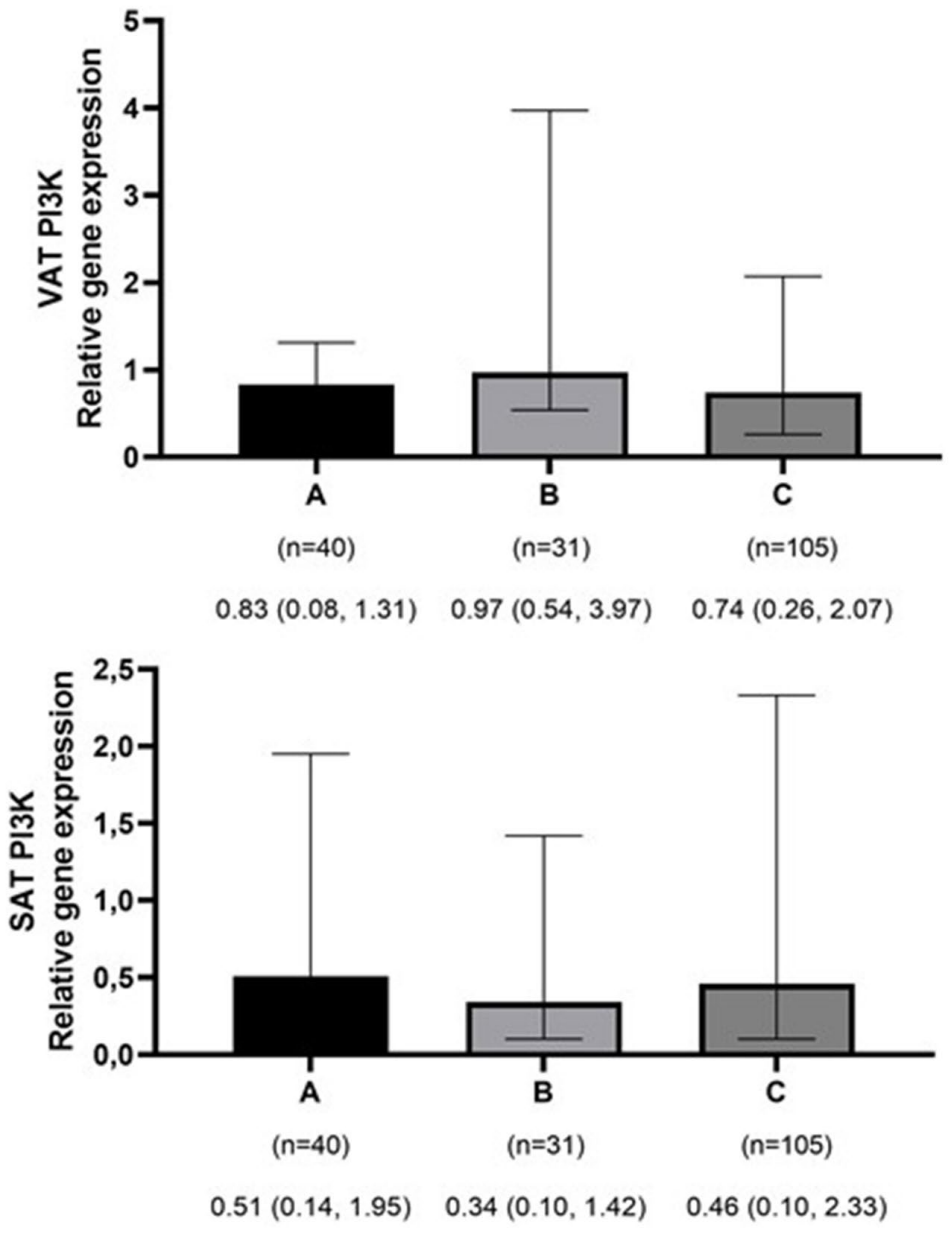
Comparison of relative expression of PI3K gene in visceral (VAT) and subcutaneous adipose tissues (SAT) among normal weight **(A)**, overweight **(B)**, and obese patients **(C)**. The numbers below the figure indicates the number of participants and the median (Interquartile ranges 25–75%). *p-value for VAT PI3K < 0.001.

**Figure 2:**
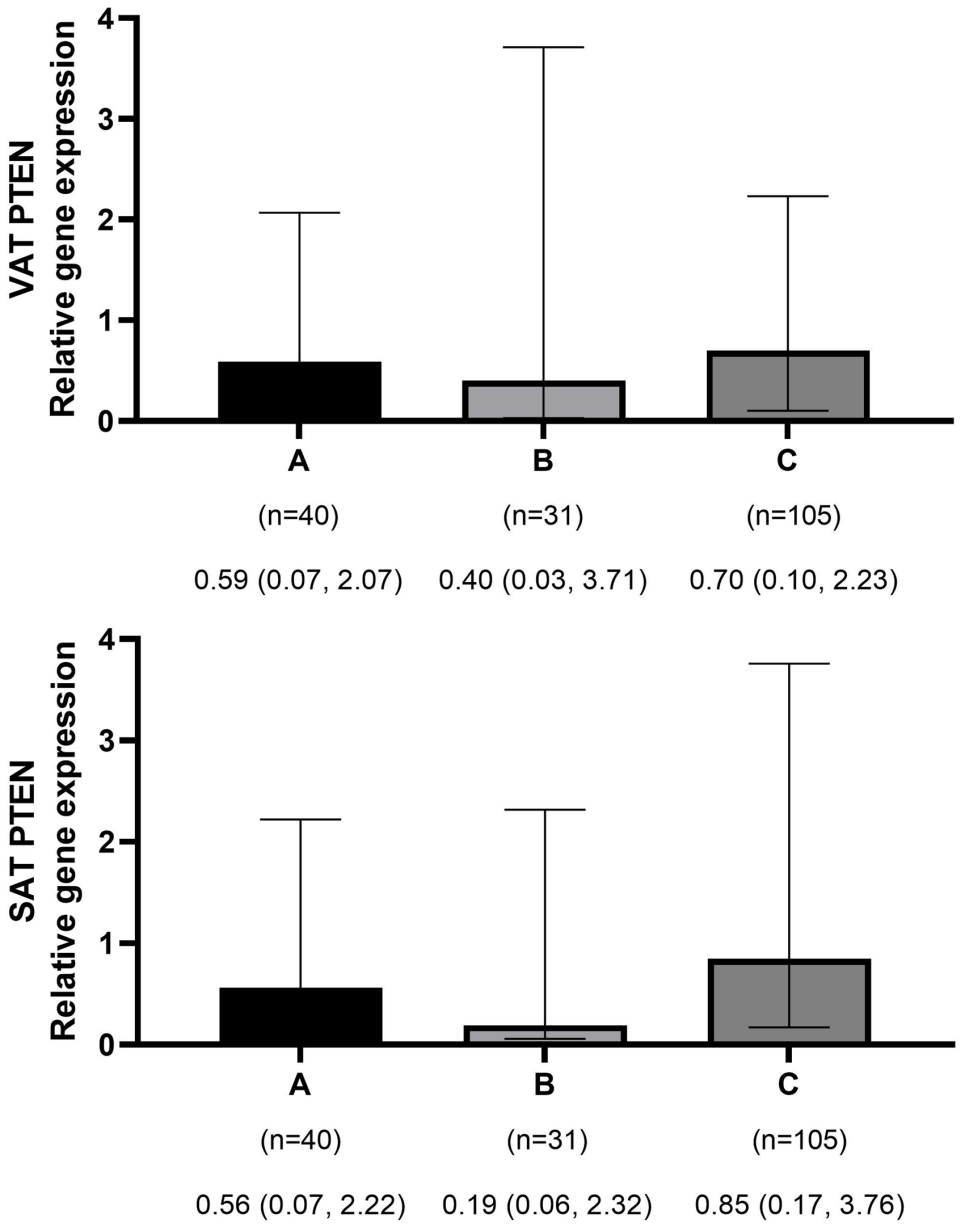
Comparison of relative expression of PTEN gene in visceral (VAT) and subcutaneous adipose tissues (SAT) among normal weight **(A)**, overweight **(B)**, and obese patients **(C)**. The numbers below the figure indicates the number of participants and the median (Interquartile ranges 25–75%).

**Figure 3:**
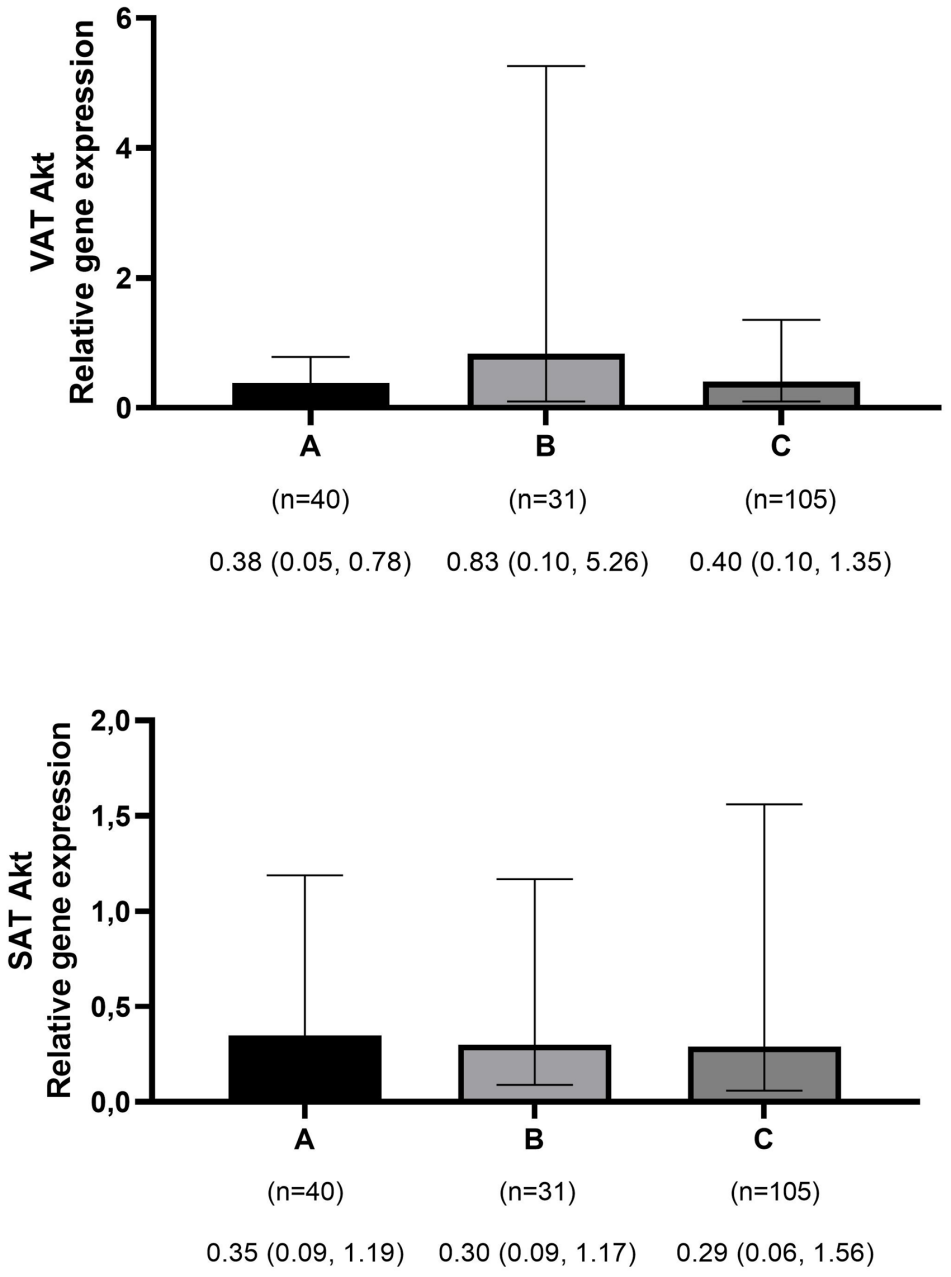
Comparison of relative expression of Akt gene in visceral (VAT) and subcutaneous adipose tissues (SAT) among normal weight **(A)**, overweight **(B)**, and obese patients **(C)**. The numbers below the figure indicates the number of participants and the median (Interquartile ranges 25–75%).

The mean ± SD age and BMI of the participants were 41.3 ± 13.6 years and 35.6 ± 10.3 kg/m^2^, respectively. The majority of the study population was women, comprising 75.6%, while men constituted 24.4%. The data on the general characteristics of individuals according to BMI classification including normal weight, overweight, and obese are shown in Table 2. Based on the findings of Table 2, the individuals who were in the obese group significantly had higher scores of GL, IL, EDIH, EDIR, ELIH, ELIR, LIH, and LIR compared to those with normal BMI (*p*-value < 0.05); however, individuals in the obese group had a lower age, II scores, DIR score, and expression of visceral PI3K (Figure 1) gene compared to those with normal BMI (*p*-value < 0.05). No significant differences were observed in other variables among the three above-mentioned groups (Figures 2, 3).

**Table 2:** Demographic characteristics of the participants based on their body mass index status.^*^

	Normal-weight patients (*n* = 40)	Overweight patients (*n* = 31)	Obese patients (*n* = 105)	*p*-value**
Age (y)	47.1 ± 15.2	49.8 ± 14.6	37.0 ± 6.1	**<0.001**
Male (%)	11 (27.5)	11 (35.5)	21 (20.0)	0.197
Physical activity (MET/h/week)	1,047 (357, 2,653)	1,350 (792, 3,897)	768 (319, 1,644)	0.061
Body mass index (kg/m^2^)	22.6 ± 1.7	27.2 ± 1.3	43.0 ± 6.1	**<0.001**
Insulinemic indices				
Glycemic index	58.7 ± 7.4	58.1 ± 6.8	58.9 ± 6.9	0.887
Glycemic load	190.6 ± 67.1	223.2 ± 72.8	261.1 ± 110.6	**<0.001**
Insulin index	57.3 ± 7.4	57.1 ± 4.5	55.2 ± 4.86	**0.022**
Insulin load	247.5 ± 67.3	296.4 ± 115.8	333.4 ± 131.2	**0.001**
EDIH	0.12 (0.01, 0.22)	0.08 (−0.02, 0.21)	0.23 (0.08, 0.38)	**0.004**
EDIR	0.57 (0.39, 0.74)	0.69 (0.37, 1.09)	0.77 (0.54, 1.17)	**0.004**
ELIH	0.12 (−1.49, 0.74)	−0.04 (−2.48, 0.57)	1.54 (0.59, 1.95)	**<0.001**
ELIR	2.43 (0.52, 4.29)	2.34 (0.15, 3.72)	4.40 (3.12, 6.27)	**<0.001**
LIH	−56.3 (−158.6, −12.3)	−73.7 (−235.7, −37.9)	−26.3 (−83.2, 2.37)	**0.004**
LIR	−56.3 (−158.5, −12.3)	−73.6 (−235.6, −37.8)	−26.1 (−83.0, 2.63)	**0.004**
DIH	0.07 (0.01, 0.15)	0.02 (−0.01, 0.17)	0.02 (0.00, 0.09)	0.091
DIR	−0.02 (−0.06, 0.02)	−0.05 (−0.09, 0.04)	−0.06 (−0.12, −0.00)	**0.019**

DIH, dietary index for hyperinsulinemia; DIR, dietary index for insulin resistance; EDIH, empirical dietary index for hyperinsulinemia; EDIR, empirical dietary index for insulin resistance; ELIH, empirical lifestyle index for hyperinsulinemia; ELIR, empirical lifestyle index for insulin resistance; LIH, lifestyle index for hyperinsulinemia; LIR, lifestyle index for insulin resistance.

* Data are presented as mean (± standard deviation) and median (25–75 interquartile range).

** Bold values indicate statistical significance *p* < 0.05.

### Insulinemic indices and genes expression in visceral adipose tissue

Table 3 indicates the relationship between different dietary insulinemic indices with the expression of PI3K, PTEN, and Akt genes in VAT among patients undergoing abdominal surgery. In normal-weight patients, there was an inverse relationship between LIH (*B* = −0.00; 95%CI: −0.00 to −0.00, *p*-value = 0.045) and LIR (*B* = −0.00; 95%CI: −0.00 to −0.00, *p*-value = 0.045) with the expression of PI3K gene. Moreover, an inverse association was observed between IL (*B* = −0.00; 95%CI: −0.01 to −0.00, *p*-value = 0.032) with the expression of the Akt gene; however, we observed a direct association between GI and the expression of PTEN (*B* = 0.04; 95%CI: 0.00–0.08, *p*-value = 0.021). Based on Table 3, in overweight patients, higher IL (*B* = 0.00; 95%CI: 0.00–0.01, *p*-value = 0.049) and DIR (*B* = 4.96; 95%CI: 1.96–7.95, *p*-value = 0.002) scores were associated with the higher expression of the PTEN gene. The association of other dietary glycemic and insulinemic indices (GI, GL, II, EDIH, EDIR, ELIH, ELIR, LIH, LIR, and DIH) with the expression of PTEN was not significant. Furthermore, no significant association was found between all dietary glycemic and insulinemic indices and the expression of PI3K and Akt genes in VAT. Table 3 also indicates that, among the obese group, there were positive associations between LIH (*B* = 0.00; 95%CI: 0.00–0.00, *p*-value = 0.045) and LIR (*B* = 0.00; 95%CI: 0.00–0.00, *p*-value = 0.045) and the expression of PI3K gene. Moreover, there was a positive association between ELIH (*B* = 0.13; 95%CI: 0.03–0.23, *p*-value = 0.009), ELIR (*B* = 0.09; 95%CI: 0.02–0.16, *p*-value = 0.012), LIH (*B* = 0.00; 95%CI: 0.00–0.00, *p*-value = 0.024), and LIR (*B* = 0.00; 95%CI: 0.00–0.00, *p*-value = 0.024) and the expression of PTEN gene. Furthermore, we observed a direct association between EDIH (*B* = 0.96; 95% CI: 0.16–1.75, *p*-value = 0.018), ELIH (*B* = 0.11; 95% CI: 0.01–0.21, *p*-value = 0.026), LIH (*B* = 0.00; 95% CI: 0.00–0.00, *p*-value = 0.047), and LIR (*B* = 0.00; 95% CI: 0.00–0.00, *p*-value = 0.047) and the expression of Akt gene.

**Table 3:** The association of dietary glycemic and insulinemic indices with expression of PI3K, PTEN, and Akt genes in visceral fat tissue of patients undergoing abdominal surgery.

	PI3K		PTEN		Akt	
	*β* (95%CI)^*^	*p*-value^**^	*β* (95%CI)^*^	*p*-value^**^	*β* (95%CI)^*^	*p*-value^**^
Normal weight (*n* = 40)						
Glycemic index	0.00 (−0.03 to 0.05)	0.776	0.04 (0.00 to 0.08)	**0.021**	−0.01 (−0.05 to 0.03)	0.618
Glycemic load	−0.00 (−0.01 to 0.00)	0.604	0.00 (−0.00 to 0.01)	0.678	−0.00 (−0.01 to 0.00)	0.203
Insulin index	−0.03 (−0.11 to 0.04)	0.404	0.01 (−0.05 to 0.09)	0.619	−0.03 (−0.11 to 0.04)	0.389
Insulin load	−0.00 (−0.14 to 0.00)	0.263	−0.00 (−0.01 to 0.00)	0.263	−0.00 (−0.01 to −0.00)	**0.032**
EDIH	2.14 (−0.42 to 4.72)	0.099	1.98 (−0.47 to 4.44)	0.110	0.22 (−2.50 to 2.95)	0.867
EDIR	−0.13 (−1.31 to 1.03)	0.811	0.01 (−1.00 to 1.03)	0.982	−0.46 (−1.64 to 0.72)	0.431
ELIH	−0.08 (−0.16 to 0.00)	0.054	−0.06 (−0.14 to 0.01)	0.113	−0.05 (−0.14 to 0.03)	0.221
ELIR	−0.06 (−0.13 to 0.01)	0.110	−0.03 (−0.11 to 0.03)	0.264	−0.04 (−0.11 to 0.03)	0.306
LIH	−0.00 (−0.00 to −0.00)	**0.045**	−0.00 (−0.00 to 0.00)	0.096	−0.00 (−0.00 to 0.00)	0.213
LIR	−0.00 (−0.00 to −0.00)	**0.045**	−0.00 (−0.00 to 0.00)	0.096	−0.00 (−0.00 to 0.00)	0.213
DIH	0.86 (−3.65 to 5.37)	0.700	1.79 (−2.23 to 5.83)	0.370	−1.74 (−6.51 to 3.02)	0.459
DIR	−1.81 (−8.35 to 4.71)	0.573	0.46 (−5.35 to 6.27)	0.872	−4.92 (−11.8 to 2.01)	0.157
Overweight (*n* = 31)						
Glycemic index	0.00 (−0.05 to 0.06)	0.788	0.02 (−0.03 to 0.08)	0.397	−0.00 (−0.06 to 0.06)	0.938
Glycemic load	0.00 (−0.00 to 0.01)	0.646	0.00 (−0.00 to 0.01)	0.225	0.00 (−0.01 to 0.01)	0.810
Insulin index	0.00 (−0.09 to 0.10)	0.909	0.04 (−0.05 to 0.13)	0.384	−0.00 (−0.10 to 0.10)	0.962
Insulin load	0.00 (−0.00 to 0.01)	0.096	0.00 (0.00 to 0.01)	**0.049**	0.00 (−0.00 to 0.01)	0.217
EDIH	−0.68 (−2.37 to 1.00)	0.413	−1.16 (−2.77 to 0.44)	0.147	−0.62 (−2.66 to 1.42)	0.533
EDIR	0.92 (−0.20 to 2.04)	0.104	0.95 (−0.14 to 2.05)	0.084	0.89 (−0.37 to 2.18)	0.160
ELIH	−0.01 (−0.08 to 0.06)	0.755	0.00 (−0.07 to 0.07)	0.990	0.01 (−0.07 to 0.09)	0.804
ELIR	0.00 (−0.06 to 0.08)	0.814	0.02 (−0.05 to 0.09)	0.503	0.03 (−0.05 to 0.12)	0.441
LIH	−0.00 (−0.00 to 0.00)	0.745	−0.00 (−0.00 to 0.00)	0.995	0.00 (−0.00 to 0.00)	0.806
LIR	−0.00 (−0.00 to 0.00)	0.745	−0.00 (−0.00 to 0.00)	0.995	0.00 (−0.00 to 0.00)	0.806
DIH	1.29 (−1.82 to 4.41)	0.400	−0.20 (−3.73 to 3.32)	0.903	1.30 (−2.00 to 4.59)	0.421
DIR	2.41 (−1.18 to 6.00)	0.179	4.96 (1.96–7.95)	**0.002**	2.42 (−1.41 to 6.26)	0.203
Obese (*n* = 105)						
Glycemic index	0.00 (−0.02 to 0.03)	0.837	−0.00 (−0.03 to 0.02)	0.959	−0.00 (−0.03 to 0.02)	0.887
Glycemic load	−0.00 (−0.00 to 0.00)	0.905	0.00 (−0.00 to 0.00)	0.740	−0.00 (−0.00 to 0.00)	0.233
Insulin index	−0.03 (−0.07 to 0.00)	0.119	0.01 (−0.02 to 0.05)	0.561	−0.02 (−0.06 to 0.01)	0.225
Insulin load	−0.00 (−0.00 to 0.00)	0.647	0.00 (−0.00 to 0.00)	0.333	−0.00 (−0.00 to 0.00)	0.606
EDIH	0.84 (−0.04 to 1.73)	0.064	0.49 (−0.32 to 1.30)	0.236	0.96 (0.16 to 1.75)	**0.018**
EDIR	0.04 (−0.33 to 0.42)	0.803	0.06 (−0.31 to 0.44)	0.726	0.18 (−0.18 to 0.56)	0.322
ELIH	0.09 (0.00 to 0.19)	0.051	0.13 (0.03 to 0.23)	**0.009**	0.11 (0.01 to 0.21)	**0.026**
ELIR	0.06 (−0.01 to 0.13)	0.087	0.09 (0.02 to 0.16)	**0.012**	0.06 (−0.00 to 0.14)	0.063
LIH	0.00 (0.00 to 0.00)	**0.045**	0.00 (0.00 to 0.00)	**0.024**	0.00 (0.00 to 0.00)	**0.047**
LIR	0.00 (0.00 to 0.00)	**0.045**	0.00 (0.00 to 0.00)	**0.024**	0.00 (0.00 to 0.00)	**0.047**
DIH	−0.43 (−2.92 to 2.05)	0.730	0.05 (−2.33 to 2.44)	0.963	−1.34 (−3.80 to 1.12)	0.283
DIR	0.66 (−1.65 to 2.98)	0.570	0.27 (−2.03 to 2.57)	0.816	−0.75 (−3.07 to 1.55)	0.518

DIH, dietary index for hyperinsulinemia; DIR, dietary index for insulin resistance; EDIH, empirical dietary index for hyperinsulinemia; EDIR, empirical dietary index for insulin resistance; ELIH, empirical lifestyle index for hyperinsulinemia; ELIR, empirical lifestyle index for insulin resistance; LIH; lifestyle index for hyperinsulinemia; LIR, lifestyle index for insulin resistance.

* Adjusted for age, sex, and energy intake.

** ABold values indicate statistical significance *p* < 0.05.

### Insulinemic indices and gene expression in subcutaneous adipose tissue

The association between different dietary insulinemic indices with gene expression of PI3K, PTEN, and Akt genes in SAT among patients undergoing abdominal surgery is shown in Table 4. In the normal-weight and overweight patients, no significant association was observed between dietary glycemic and insulinemic indices and the expression of PI3K, PTEN, and Akt genes in SAT. However, in the obese group, there was a positive association between EDIH and the expression of PI3K (*B* = 1.39; 95% CI: 0.62–2.16, *p*-value = 0.001), PTEN (*B* = 1.05; 95% CI: 0.30–1.80, *p*-value = 0.006), and Akt (*B* = 1.05; 95% CI: 0.24–1.87, *p*-value = 0.012) genes. The association of other dietary glycemic and insulinemic indices with the genes mentioned above was not significant.

**Table 4:** The association of dietary glycemic and insulinemic indices with expression of PI3K, PTEN, and Akt genes in subcutaneous fat tissue of patients undergoing abdominal surgery.

	PI3K		PTEN		Akt	
	*β* (95%CI)^*^	*p*-value^**^	*β* (95%CI)^*^	*p*-value^**^	*β* (95%CI)^*^	*p*-value^**^
Normal weight (*n* = 40)						
Glycemic index	0.00 (−0.03 to 0.05)	0.650	−0.00 (−0.04 to 0.04)	0.971	−0.02 (−0.06 to 0.02)	0.303
Glycemic load	0.00 (−0.00 to 0.01)	0.843	0.00 (−0.00 to 0.00)	0.855	0.00 (−0.01 to 0.00)	0.496
Insulin index	−0.01 (−0.09 to 0.06)	0.756	−0.01 (−0.08 to 0.05)	0.652	−0.01 (−0.08 to 0.06)	0.798
Insulin load	−0.00 (−0.01 to 0.00)	0.692	−0.00 (−0.10 to 0.00)	0.540	−0.00 (−0.01 to 0.00)	0.288
EDIH	2.04 (−0.30 to 4.38)	0.086	0.16 (−2.27 to 2.60)	0.890	−0.82 (−3.35 to 1.69)	0.507
EDIR	0.27 (−0.76 to 1.30)	0.598	−0.38 (−1.34 to 0.57)	0.418	−0.29 (−1.37 to 0.79)	0.589
ELIH	0.00 (−0.08 to 0.10)	0.849	−0.01 (−0.09 to 0.06)	0.783	0.00 (−0.07 to 0.09)	0.862
ELIR	0.01 (−0.06 to 0.09)	0.718	−0.01 (−0.08 to 0.05)	0.586	0.00 (−0.07 to 0.07)	0.889
LIH	0.00 (−0.00 to 0.00)	0.890	0.00 (−0.00 to 0.00)	0.789	0.00 (−0.00 to 0.00)	0.835
LIR	0.00 (−0.00 to 0.00)	0.890	0.00 (−0.00 to 0.00)	0.789	0.00 (−0.00 to 0.00)	0.835
DIH	2.14 (−1.75 to 6.03)	0.270	−0.67 (−4.55 to 3.21)	0.726	−0.57 (−4.65 to 3.49)	0.773
DIR	1.37 (−4.32 to 7.07)	0.625	1.16 (−4.35 to 6.68)	0.669	0.24 (−5.80 to 6.28)	0.935
Overweight (*n* = 31)						
Glycemic index	−0.04 (−0.10 to 0.02)	0.171	−0.04 (−0.10 to 0.01)	0.124	−0.04 (−0.10 to 0.02)	0.185
Glycemic load	−0.00 (−0.01 to 0.00)	0.280	−0.00 (−0.01 to 0.00)	0.184	−0.00 (−0.01 to 0.00)	0.231
Insulin index	0.03 (−0.07 to 0.14)	0.519	0.05 (−0.05 to 0.15)	0.335	0.01 (−0.09 to 0.12)	0.750
Insulin load	0.00 (−0.00 to 0.01)	0.902	−0.00 (−0.01 to 0.00)	0.567	−0.00 (−0.01 to 0.00)	0.665
EDIH	−0.06 (−1.96 to 1.83)	0.944	0.74 (−1.11 to 2.59)	0.417	0.11 (−1.79 to 2.03)	0.900
EDIR	−0.58 (−1.88 to 0.70)	0.358	0.04 (−1.26 to 1.35)	0.948	0.12 (−1.20 to 1.46)	0.844
ELIH	0.03 (−0.04 to 0.11)	0.376	0.01 (−0.06 to 0.09)	0.564	0.02 (−0.05 to 0.10)	0.535
ELIR	0.01 (−0.07 to 0.09)	0.782	0.00 (−0.08 to 0.08)	0.999	0.01 (−0.07 to 0.09)	0.781
LIH	0.00 (−0.00 to 0.00)	0.384	0.00 (−0.00 to 0.00)	0.648	0.00 (−0.00 to 0.00)	0.530
LIR	0.00 (−0.00 to 0.00)	0.384	0.00 (−0.00 to 0.00)	0.648	0.00 (−0.00 to 0.00)	0.530
DIH	−2.02 (−5.41 to 1.36)	0.229	−1.91 (−5.29 to 1.45)	0.251	−1.22 (−4.72 to 2.28)	0.478
DIR	−1.43 (−5.53 to 2.65)	0.475	−2.22 (−6.21 to 1.76)	0.261	−0.27 (−4.45 to 3.91)	0.895
Obese (*n* = 105)						
Glycemic index	0.01 (−0.01 to 0.04)	0.246	0.00 (−0.02 to 0.02)	0.953	0.01 (−0.01 to 0.03)	0.478
Glycemic load	0.00 (−0.00 to 0.00)	0.748	−0.00 (−0.00 to 0.00)	0.691	0.00 (−0.00 to 0.00)	0.849
Insulin index	−0.04 (−0.08 to 0.00)	**0.037**	−0.01 (−0.05 to 0.02)	0.421	−0.03 (−0.07 to 0.00)	0.075
Insulin load	−0.00 (−0.00 to 0.00)	0.594	0.00 (−0.00 to 0.00)	0.343	−0.00 (−0.00 to 0.00)	0.644
EDIH	1.39 (0.62 to 2.16)	**0.001**	1.05 (0.30 to 1.80)	**0.006**	1.05 (0.24 to 1.87)	**0.012**
EDIR	0.31 (−0.05 to 0.69)	0.092	0.18 (−0.17 to 0.54)	0.313	0.22 (−0.15 to 0.60)	0.246
ELIH	0.00 (−0.10 to 0.10)	0.990	0.06 (−0.03 to 0.15)	0.201	−0.17 (−0.11 to 0.08)	0.746
ELIR	0.03 (−0.04 to 0.10)	0.375	0.03 (−0.03 to 0.10)	0.287	0.02 (−0.04 to 0.10)	0.478
LIH	−0.00 (−0.00 to 0.00)	0.920	0.00 (−0.00 to 0.00)	0.186	0.00 (−0.00 to 0.00)	0.851
LIR	−0.00 (−0.00 to 0.00)	0.920	0.00 (−0.00 to 0.00)	0.186	0.00 (−0.00 to 0.00)	0.851
DIH	−1.32 (−3.70 to 1.04)	0.270	−0.16 (−2.43 to 2.10)	0.886	−1.30 (−3.74 to 1.13)	0.291
DIR	−1.29 (−3.56 to 0.97)	0.259	0.00 (−2.18 to 2.19)	0.998	−1.06 (−3.36 to 1.24)	0.363

DIH, dietary index for hyperinsulinemia; DIR, dietary index for insulin resistance; EDIH, empirical dietary index for hyperinsulinemia; EDIR, empirical dietary index for insulin resistance; ELIH, empirical lifestyle index for hyperinsulinemia; ELIR, empirical lifestyle index for insulin resistance; LIH, lifestyle index for hyperinsulinemia; LIR, lifestyle index for insulin resistance.

* Adjusted for age, sex, and energy intake.

** Bold values indicate statistical significance *p* < 0.05.

## Discussion

In the current study, for the first time, the association between different dietary glycemic and insulinemic indices (GI, GL, II, IL, EDIH, EDIR, ELIH, ELIR, DIH, DIR, LIH, and LIR) with the PI3K, PTEN, and Akt gene expressions in VAT and SAT in three groups of participants (normal-weight, overweight, and obese) has been investigated. In normal-weight participants, we found a positive association between dietary GI and expression of the PTEN gene; however, we observed an inverse association between LIH and LIR scores and PI3K expression as well as an inverse association between IL and expression of the Akt gene in VAT. In overweight patients, there is a positive association between IL and DIR scores and PTEN gene expression in VAT. Moreover, in obese patients, we found a positive association between ELIH, LIH, and LIR and PI3K gene expression, a positive association between ELIH, ELIR, LIH, and LIR and PTEN gene expression, and a positive association between EDIH, ELIH, LIH, and LIR and Akt gene expression in VAT. In SAT, there was a direct association between EDIH and the expression of PI3K, PTEN, and Akt genes and an inverse association between II and the expression PI3K gene among the obese group.

To the best of our knowledge, the present study is the first study to assess the association between insulinemic indices and the expression of those genes involved in insulin action such as PI3K, PTEN, and Akt. However, some studies have focused on certain aspects of diet quality on insulin metabolism or its related gene expression. Our findings on the association between dietary glycemic and insulinemic indices and PI3K, PTEN, and Akt gene expressions among participants are comparable to the results of the previously published experimental studies or observational human studies reporting that high insulinemic and glycemic potential of diet is positively associated with the expression of PI3K and Akt genes (35, 36). High-fat and calorie restriction diets as low insulinemic diets have a minor role in the stimulation of insulin from *β*-cells (21–23). These diets inhibit the PI3K/Akt pathway and decrease glucose transport into the cell by decreasing the glucose transporter type 4 (GLUT4) in the plasma membrane (22, 23). Other animal studies have shown a high carbohydrate diet activates the PI3K/Akt signaling pathway (36). Moreover, several studies have evaluated the association between various indices and food groups with the mentioned gene expression in both humans and animals, revealing controversial findings (19, 20, 37–39). Several animal studies indicated food groups such as high starch intake, whole grain diet, and whole grain cereal intake stimulated the expression and activation of the PI3K/Akt pathway (19, 20, 38). These results are in line with our findings among obese participants that higher insulinemic indices were related to higher expression of PI3K, Akt, and even PTEN. It seems that the insulinogenic diet among these individuals, activated the PI3K/Akt signaling to induce *de novo* lipogenesis by increased activation and expression of the transcription factor sterol regulatory element-binding protein-1 (SREBP1) (40). However, the increase in PTEN expression—a negative regulator of the PI3K/Akt pathway—associated with higher dietary insulinemic indices in our study of obese adults can be justified by PTEN’s role as a co-regulator of both lipid metabolism and oncogenesis (36, 41). Therefore, prolonged increases in insulin secretion lead to simultaneous activation of PTEN along with the PI3K/Akt pathway. This activation helps prevent excess fat storage and inhibits other effects of insulin that could lead to the over-proliferation of cell precursors and cancerous cells (42). Indeed, PTEN acts as a check valve to prevent aberrant growth that can potentially be caused by hyperinsulinemia (41). In line with the regulatory effects of PTEN, a previous observational study found no association between plasma glucose and insulin units administered with the expression of PTEN; however, total energy intake administered and expression of PTEN were positively correlated (43). In addition, another cross-sectional study by Kadkhoda et al. reported that the expression of PTEN was positively associated with fruit intake in VAT and SAT of obese participants (37). Although fruit intake generally has a minimal effect on insulin secretion stimulated by fructose (fruit sugar) (44), high fruit consumption in obese people may still lead to the stimulation of PTEN expression. This response helps control the insulin secretion and cellular function due to the high consumption of calories.

On the other hand, our findings in this investigation indicate an inverse association between insulinogenic and induced IR by diet (LIH and LIR) with the expression of PI3K and also between IL and Akt genes in VAT, among participants with normal weight. This inverse relationship could be due to higher serum glucose uptake by the liver, muscle tissue, and brain cells in these normal-weight subjects and there is no need to store the glucose and other macronutrient as fat storage in the VAT. In other words, less glucose is available to enter fat tissue cells and *de novo* lipogenesis among normal-weight participants. However, among people who are metabolically obese but of normal weight, despite their normal weight, there is evidence of reduced insulin sensitivity, increased abdominal adiposity, and a more atherogenic lipid profile (45).

The findings of the present study to a large extent indicate the mechanisms of the cellular effect of insulin through the PI3K/Akt pathway in storing excess calorie intake received by a higher insulinotropic diet as fat in the visceral tissues of overweight and obese people. Insulin binds to its receptors on the surface of adipose tissue cells and activates the insulin receptor substrates 1 and 2 (IRS-1/2). The activated PI3K by the IRS-1/2 results in the phosphorylation of PIP2 to PIP3. PIP3 increased the expression of the Akt gene and its activity to promote glucose uptake by facilitating the translocation of GLUT4 to the adipose cell membrane. Then, excessive glucose uptake is stored as fat in adipose tissue (46).

The present study had some strengths. First, it was the first study to provide valuable evidence on different dietary glycemic and insulinemic indices and their association with the expression of PI3K, PTEN, and Akt genes in adipose tissue among individuals in three BMI categories. Second, this relationship was assessed using 12 dietary glycemic and insulinemic indices, some of which were developed and validated among the Iranian population. Third, we used a validated semi-quantitative FFQ to calculate the dietary data, which minimized measurement errors. Some limitations of this research need to be mentioned. The present investigation had a cross-sectional design, so we could not show a causal association. Another limitation was that the Iranian FCT is not complete in some food items, so we had to use the USDA FCT to calculate participants’ energy intakes; however, the majority of FFQ items were common food and only for breads which were different in Iranian FCT. We reviewed all bread types in the USDA FCT and chose those most similar to the breads consumed by our population to eliminate the potential limitations due to differences in bread types. Finally, despite controlling potentially confounding variables, other possible confounders such as race, serum hormonal status, and familial genetic predispositions may still affect the relationship between dietary insulinemic indices and expression of PI3K, PTEN, and Akt genes.

## Conclusion

Our findings revealed that high insulinemic dietary patterns may be related to PI3K, PTEN, and Akt expression in adipose tissue. These results were more significant and stronger in obese individuals; in this group, a positive relationship between higher scores of insulinemic indices, indicating hyperinsulinemic and induced IR by diet, and the expression of PI3K, PTEN, and Akt was found. Moreover, in overweight participants, there was a positive association between IL and DIR scores and PTEN gene expression in VAT. Furthermore, among individuals with normal weight, a hyperinsulinemic diet, defined by higher IL, LIH, and LIR scores, was associated with decreased expression of PI3K and Akt genes and increased PTEN expression in VAT.

## Data Availability

The raw data supporting the conclusions of this article will be made available by the authors, without undue reservation.
