# Supplementary material for: The association of dietary insulinemic indices with PI3K, PTEN, and Akt gene expressions in visceral and subcutaneous adipose tissues among individuals undergoing abdominal surgery
Source: Front Nutr. 2024 Oct 4;11:1467686. doi: 10.3389/fnut.2024.1467686 (PMC11519925; doi:10.3389/fnut.2024.1467686)
Supplement: Supplementary file 1 [file Table_1.docx]

**Supplementary Table 1.** The food group components and their coefficients as the weight of each component to the total score of included insulinemic indices in the study.

|  | **EDIH** | **ELIH** | **EDIR** | **ELIR** | **DIH** | **DIR** | **LIH** | **LIR** |
| --- | --- | --- | --- | --- | --- | --- | --- | --- |
| **Red meat** | +0.250 | +0.089 | +0.328 | +0.181 | -0.059 | -0.067 |  |  |
| **Low energy beverages** | +0.053 |  | +0.116 | +0.051 |  |  |  |  |
| **Cream soups** | +0.787 | +0.536 | +0.519 |  |  |  |  |  |
| **Processed meat** | +0.199 |  | +0.327 | +0.124 |  |  |  |  |
| **Margarine** | +0.054 | +0.041 | +0.121 | +0.099 |  |  |  |  |
| **poultry** | +0.183 |  |  |  |  |  |  |  |
| **Butter** | +0.094 | +0.058 |  |  |  |  |  |  |
| **French fries** | +0.581 |  |  |  |  |  |  |  |
| **Other fish** | +0.172 |  | +0.155 |  |  |  |  |  |
| **High energy beverages** | +0.104 |  |  |  |  |  |  |  |
| **Tomatoes** | +0.095 |  | +0.145 | +0.135 |  |  |  |  |
| **Low fat dairy** | +0.025 |  |  |  |  | -0.061 | -0.051 | -0.063 |
| **Eggs** | +0.124 |  |  |  |  |  |  |  |
| **Wine** | -0.165 | -0.071 | -0.261 | -0.171 |  |  |  |  |
| **Coffee** | -0.035 | -0.020 | -0.070 | -0.041 |  |  |  |  |
| **Whole fruits** | -0.029 | -0.029 |  |  |  |  |  |  |
| **High fat dairy** | -0.046 | -0.054 | -0.066 | -0.064 | -0.063 | -0.073 | -0.067 | -0.069 |
| **Green leafy vegetables** | -0.055 |  | -0.076 | -0.064 |  |  |  |  |
| **Liquor** |  | +0.072 | -0.204 | -0.122 |  |  |  |  |
| **Fruit juice** |  | +0.042 | +0.052 | +0.068 |  |  |  |  |
| **Snacks** |  | -0.024 |  |  |  | -0.055 |  |  |
| **Salad dressing** |  | -0.059 |  |  |  |  |  |  |
| **Refined grains** |  |  | +0.102 | +0.076 | +0.105 | +0.079 | +0.091 | +0.077 |
| **Other vegetables** |  |  | +0.126 | +0.070 |  |  |  |  |
| **Beer** |  |  | -0.210 |  |  |  |  |  |
| **Dark yellow vegetables** |  |  | -0.103 |  |  |  |  |  |
| **Nuts** |  |  | -0.078 |  |  |  |  |  |
| **potatoes** |  |  |  | +0.160 |  |  |  |  |
| **Tea** |  |  |  | +0.027 |  |  |  |  |
| **Pickles** |  |  |  |  | +0.089 | +0.085 |  |  |
| **Doogh** |  |  |  |  | +0.082 | +0.066 | +0.076 | +0.061 |
| **Sweetened beverages** |  |  |  |  | +0.062 | +0.059 |  |  |
| **Fish** |  |  |  |  | +0.061 | +0.056 | +0.044 |  |
| **Lemon juice** |  |  |  |  | +0.061 | +0.063 |  |  |
| **Broth** |  |  |  |  | -0.059 | -0.061 |  |  |
| **Starchy vegetables** |  |  |  |  | -0.068 | -0.054 | -0.084 | -0.065 |
| **Body mass index** |  | +0.051 |  | +0.047 |  |  | +0.482 | +0.487 |
| **Physical activity** |  | -0.001 |  | -0.064 |  |  | -0.064 | -0.064 |
